# Investigating the Effect of Mono- and Dimeric 360A G-Quadruplex Ligands on Telomere Stability by Single Telomere Length Analysis (STELA)

**DOI:** 10.3390/molecules24030577

**Published:** 2019-02-06

**Authors:** In Pyo Hwang, Patrick Mailliet, Virginie Hossard, Jean-Francois Riou, Anthony Bugaut, Lauréline Roger

**Affiliations:** “Structure and Instability of Genomes” Laboratory, Muséum National d’Histoire Naturelle (MNHN), Inserm U1154, CNRS UMR 7196, 43 rue Cuvier, 75005 Paris, France; in-pyo.hwang@etu.parisdescartes.fr (I.P.H.); patrick.mailliet@mnhn.fr (P.M.); virginie.hossard@mnhn.fr (V.H.); riou@mnhn.fr (J.-F.R.)

**Keywords:** G-quadruplex structures, G-quadruplex ligands, telomere, STELA

## Abstract

Telomeres are nucleoprotein structures that cap and protect the natural ends of chromosomes. Telomeric DNA G-rich strands can form G-quadruplex (or G4) structures. Ligands that bind to and stabilize G4 structures can lead to telomere dysfunctions by displacing shelterin proteins and/or by interfering with the replication of telomeres. We previously reported that two pyridine dicarboxamide G4 ligands, 360A and its dimeric analogue (360A)_2A_, were able to displace in vitro hRPA (a single-stranded DNA-binding protein of the replication machinery) from telomeric DNA by stabilizing the G4 structures. In this paper, we perform for the first time single telomere length analysis (STELA) to investigate the effect of G4 ligands on telomere length and stability. We used the unique ability of STELA to reveal the full spectrum of telomere lengths at a chromosome terminus in cancer cells treated with 360A and (360A)_2A_. Upon treatment with these ligands, we readily detected an increase of ultrashort telomeres, whose lengths are significantly shorter than the mean telomere length, and that could not have been detected by other methods.

## 1. Introduction

Telomeres are nucleoprotein structures that cap and protect the natural ends of chromosomes by preventing them from being recognized as DNA double strand breaks [1]. In human cells, telomeric DNA is composed of the TTAGGG sequence tandemly repeated to a size up to 25 kb. Their functions rely on a complex of telomere-associated-proteins named “shelterin” [2]. In most somatic cells, telomeres erode at each cell division due to the so-called “end-replication problem” [3,4]. But they can also shorten as a consequence of stochastic deletion events, resulting in telomeres extremely shorter than the bulk population, often called TDEs (for telomere deletion events) [5,6,7,8]. The underlying mechanisms that result in these TDEs are still unclear. Telomere erosion ultimately results in a partial loss of telomere function, triggering a stable cell cycle arrest termed replicative senescence. In the absence of fully functional DNA damage checkpoints, telomeres can shorten to a length at which they become dysfunctional and capable of fusion with other telomeres or with non-telomeric loci, which may contribute to the acquisition of large-scale genomic rearrangements and cancer progression.

Human telomeric G-rich sequences can form G-quadruplex (or G4) structures in vitro [9]. One of the first indications that G4 structures may be present at human telomeres came from the observation that a tritiated derivative of the selective G-quadruplex ligand 360A preferentially bound to the ends of metaphase chromosomes [10]. More recently, G4 structures at telomeres in human cells have also been visualized using an engineered structure-specific antibody [11]. Furthermore, a number of helicases, which have been shown to unwind G4 structures in vitro, localize to telomeres and are required to maintain telomere integrity [12,13]. G4 structures have also been mapped in other regions of the genome and it has been suggested that G4s may be involved in gene regulation [11,14,15].

Over the past decades, numerous small molecules have been synthesized that bind to and stabilize human telomeric G4 structures in vitro, mainly with the view to developing potential anticancer agents [16,17,18]. Some of these ligands were shown to generate telomere dysfunction in cancer cell lines, such as telomere fusion, telomere doublets, and/or telomere complete loss [19,20,21]. These telomere aberrations can result from telomere uncapping and from interference with telomere replication [22,23,24,25].

Recently, we reported on the in vitro binding properties of the pyridine dicarboxamide G4 ligand 360A and its newly synthesized dimeric analogue (360A)_2A_ to telomere-mimicking oligonucleotides forming up to four contiguous G4 structures [26]. This study revealed that both molecules stabilize telomeric higher-order G4 structures and that they were able to displace human replication protein A (hRPA) from telomeric DNA, with a greater efficiency for the dimeric ligand than the monomer form.

Here, we investigate the cellular effect of 360A and (360A)_2A_ on telomere length by undertaking single telomere length analysis (STELA). Developed by Baird et al., STELA is a PCR-based technology that gives the full spectrum of telomere lengths at a single chromosome terminus in dividing and non-dividing cells [5]. A modified version of STELA was used to identify the end nucleotide of the telomeric C-strand [27]. In one case, this STELA-based approach was used to study the effect of a bromide derivative of 360A (360A-Br) on the C-strand terminal sequence of XpYp telomere in the HT1080 fibrosarcoma cell line, which demonstrated a minor effect in the nucleotide composition [28]. However, STELA was never used to study the impact of G4 ligands on telomere length. Of particular importance for this study is the unique ability of STELA to detect ultrashort dysfunctional telomeres that cannot be visualized by other hybridization-based methods, such as telomere restriction fragment (TRF) and telomere Q-FISH. We showed that STELA is a method of choice to readily detect TDEs and that the frequency of these events tends to increase when G4 structures at telomeres are stabilized by 360A and (360A)_2A_. This work represents the first example of the use of STELA to study the effect of G4 ligands on telomere length.

## 2. Results

### 2.1. 360A and (360A)_2A_ Inhibit the Proliferation of A549 Cells with No Effect on Mean Telomere Length

**Figure 1:**
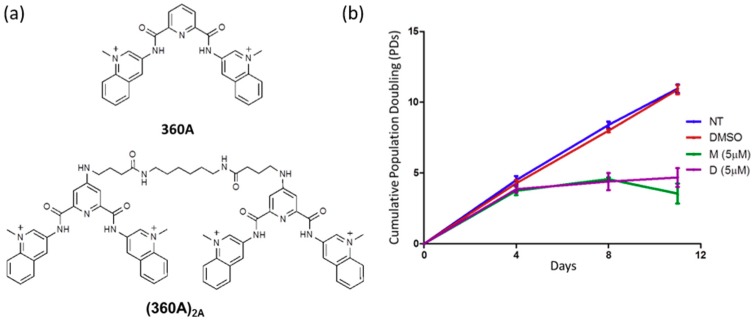
Chemical structure of 360A and (360A)_2A_ and the effect of these ligands on the proliferation of A549 cells: (**a**) Chemical structure of G4 ligands 360A and (360A)_2A_; (**b**) Cell growth curves plotting mean PDs and days in culture of non-treated A549 (NT), A549 treated with 0.1% DMSO (DMSO), with 5 μM of 360A (M), or with 5 μM of (360A)_2A_ (D) (n = 3 independent experiments; error bars, SD).

First, we evaluated the effect of 360A and its dimer form (360A)_2A_ (Figure 1a) on the cell growth of A549 lung carcinoma cell lines, a model cell line that was previously used to show the preferential binding of 360A to telomeres [10]. Cells were treated with 5 μM of 360A or (360A)_2A_ and cumulative population doublings (PDs) were calculated at each reseeding until complete growth arrest, at which point the cultures were terminated. From three independent experiments, we observed that treatment with 5 μM of 360A led to an inhibition of cell proliferation within 11 days of treatment, as previously shown in other cancer cell lines (Figure 1b) [19,20,21]. Treatment with the newly synthesized dimer (360A)_2A_ led to a comparable antiproliferative effect (Figure 1b).

**Figure 2:**
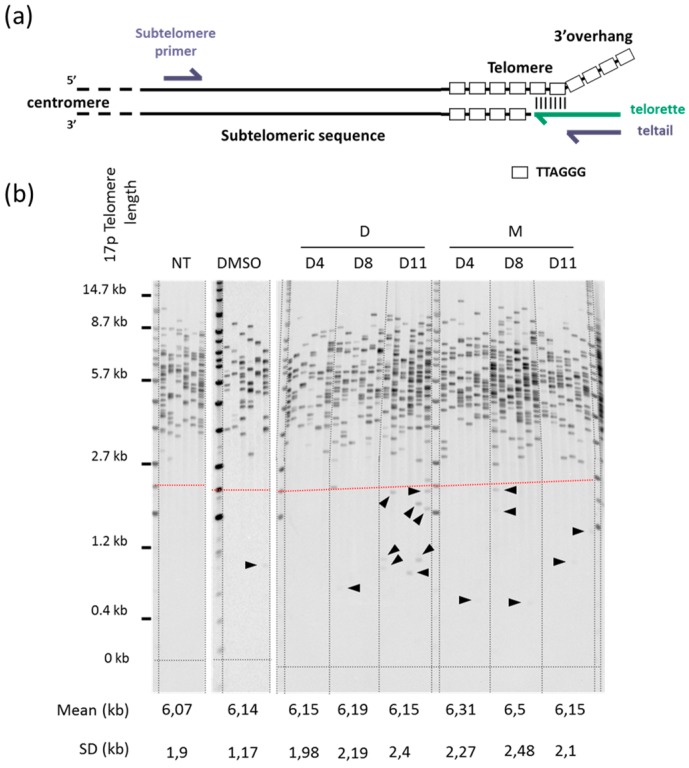
Treatment with 360A and (360A)_2A_ had no effect on mean telomere length: (**a**) Schematic representation of the principle of STELA [5]; (**b**) STELA at 17p telomere in non-treated A549 (NT), A549 treated with 0.1% DMSO (DMSO), 5 μM 360A (M), or 5 μM (360A)_2A_ (D) after 4, 8, and 11 days of treatment (D4, D8, D11) (representative STELA from experiment (1)). Each lane represents a PCR and each band a telomere. The mean and SD of the telomere length are shown below. Arrows show examples of telomere deletion events (TDEs) (i.e., telomeres shorter than 2.2 kb (red dashed line)).

Next, we used the genomic DNA extracted from samples obtained at each reseeding of the cell growth experiments to examine the effect of 360A and (360A)_2A_ on 17p (i.e., the short arm of chromosome 17) telomere length using STELA [5]. A prerequisite to STELA is the identification of a chromosome-specific telomere-adjacent DNA sequence. As the subtelomere sequence of human 17p is well characterized, STELA at 17p telomere is robust and has been extensively used [6,7,8]. STELA is a PCR-based approach that relies on the use of a set of primers comprising: (i) a linker primer, called “telorette”, constituted by seven bases complementary to the G-rich 3′ overhang followed by a unique 20-nucleotide tail non complementary to the telomere; (ii) a subtelomere specific primer close to the beginning of the telomere and specific to a chromosome end; and (iii) a primer called “teltail”, whose sequence is identical to the telorette 20-nucleotide tail (Figure 2a). Each extracted DNA sample is analyzed with typically six PCR reactions that contain between four and 30 amplifiable molecules. PCR products are then resolved on an agarose gel and detected by southern blot hybridization with a radiolabeled telomere probe. Each band detected on the membrane represents a single telomere. Thus, STELA gives the full spectrum of telomere lengths of a specific chromosome end and can detect rare short telomeres (TDEs), distinct from the bulk population, which cannot be visualized by any other methods. We performed STELA to measure 17p telomere length at each reseeding point (i.e., at day 4, 8, and 11), during the course of the cell growth experiments, in control and treated cells. Overall, we did not observe any significant difference in the mean telomere length of cells treated with 360A and (360A)_2A_ compared with control cells (non-treated or 0.1% DMSO) as a function of PDs (Figure 2b). These data are consistent with previous observations in other cancer cell lines showing that the antiproliferative effect of 2,6-pyrimide-dicarboxamide derivatives was not associated with progressive telomere shortening with ongoing cell divisions [19,21].

However, the unique ability of STELA to detect TDEs allowed us to observe rare telomeres that were significantly shorter (≤2.2 kb) than the bulk telomere length distributions (black arrow heads in Figure 2b). We noticed that these TDEs seemed more frequent in G4 ligand-treated cells than in control cells.

### 2.2. 360A and (360A)_2A_ Induce Telomere Deletion Events in A549 Cells

**Figure 3:**
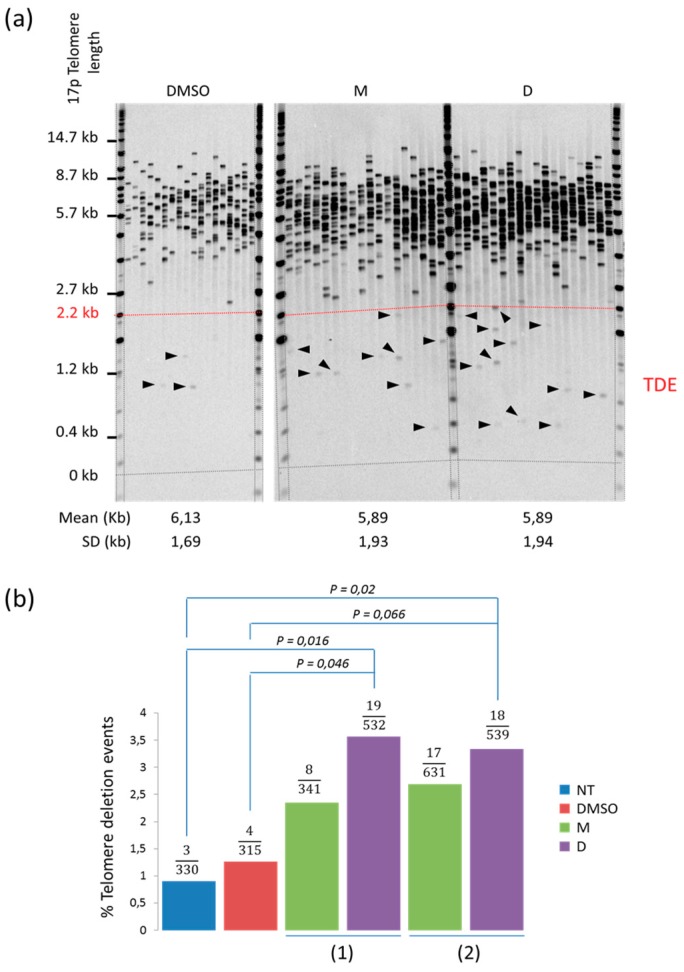
360A and (360A)_2A_ induced telomere deletion events in A549 cells: (**a**) Representative experiment (from experiment (2)) of the scaled up STELA at 17p telomere in the last PD points of A549 treated with 5 μM of 360A (M) or 5 μM of (360A)_2A_ (D). Each lane represents a PCR and each band a telomere; (**b**) Histograms showing the proportion of TDEs in 2 independent experiments in the last PD points (i.e., at day 11 for both 360A and (360A)_2A_ in experiment 1; and at day 11 and 8 for 360A and (360A)_2A_, respectively, in experiment 2) of A549 treated with 5 μM of 360A (M) or 5 μM of (360A)_2A_ (D). The Chi-square test was used to determine the p values.

In order to accurately measure the frequency of the TDEs, we next scaled up our analysis by increasing to at least 18 the number of STELA PCRs per sample (Figure 3a). The last PD points (i.e., when the cells stopped proliferating and when the cultures were terminated) of the treated cells were compared to the controls in two independent experiments. From 315 to 631 telomeres were analyzed per sample (Figure 3b). Non-treated and 0.1% DMSO cells displayed a low frequency of TDEs (0.90% and 1.27%, respectively), which was comparable to what has been previously observed at 17p telomeres in normal cells [7]. Interestingly, TDEs increased to 2.34% and 2.69% in cells treated with the G4 ligand 360A in experiment 1 and 2, respectively. Even if we observed a reproducible trend toward an increased frequency of TDEs, the differences between treated vs non-treated or 0.1% DMSO control cells were not statistically significant, probably due to the low frequency of TDEs. For the dimeric ligand (360A)_2A_, TDEs increased to 3.57% and 3.33% in experiment 1 and 2, respectively. This represents a 3.9-fold (experiment 1, chi-square test, *p* = 0.016) and 3.7-fold (experiment 2, chi-square test, *p* = 0.02) increase when compared to non-treated cells, and a 2.8-fold (experiment 1, chi-square test, *p* = 0.046) and 2.6-fold increase (experiment 2, chi-square test, *p* = 0.066) when compared to 0.1% DMSO control. Such results clearly indicate that the ligand (360A)_2A_ affects telomere stability.

## 3. Discussion

Using hybridization-based methods such as Q-FISH and TRF, many studies have showed that G4 ligands could lead to telomere shortening and complete loss. Q-FISH can inform on the telomere length of individual chromosome arms within the same cell and detect “signal free ends” called telomere loss. However, the resolution of this approach for the detection of extremely short telomeres is limited in comparison to STELA. Moreover, Q-FISH is restricted to the analysis of cells that are proliferating [29]. TRF gives the mean telomere length of all chromosomes and is biased toward the detection of longer telomeres [29].

Here, we used the unique ability of STELA to investigate, for the first time, the full spectrum of telomere lengths at a given chromosome terminus upon treatment with G4 ligands. Two pyridine dicarboxamide ligands were used: 360A and its recently reported dimer form (360A)_2A_. Our results indicate that these ligands can induce rare events of extreme telomere shortening, manifested by an increase of TDEs whose lengths are significantly shorter than the mean telomere length.

TDEs have previously been detected in telomerase negative and positive cells and in normal and cancer cells. Yet, the underlying mechanisms of TDEs in human cells are still unclear. G4s represent a barrier for the progression of replication forks, potentially leading to fork stalling or fork collapse and replication-associated DNA double strand breaks (DSBs) [13]. Many helicases are able to resolve G4 structures in vitro [30]. It has been proposed that WRN helicase is required for the replication of the G-rich telomeric strand by resolving G4s at telomeres, thus allowing the replication fork to progress [31]. Human cells lacking WRN helicase display a complete loss of telomere, specifically at the G-rich strand. So, ligand-stabilized G4s that are unresolved at telomeres could be a potential mechanism that leads to TDEs.

In our study, the frequency of TDEs appeared slightly higher in cells treated with (360A)_2A_ compared to cells treated with 360A. This is interesting in regard to our previous in vitro data showing that (360A)_2A_ displaced hRPA from telomeric G-rich DNA with a greater efficiency than 360A [26]. hRPA is a single-stranded DNA-binding protein, which is involved in DNA replication [32], and it has been shown that hRPA associates with telomeres during replication [33,34]. Interestingly, expression of a mutant RPA in HT1080 has been shown to cause telomere shortening [35]. Consequently, a greater ability of (360A)_2A_ to displace hRPA could account for the differences in TDEs frequencies between cells treated with (360A)_2A_ and 360A.

To follow, it would be interesting to extend this study to other telomeres in order to assess if the impact of our G4 ligands on telomere stability is the same across all chromosome ends. Moreover, the mechanisms underlying TDEs upon treatment with G4 ligands still need to be clarified. It would be of particular interest to investigate the displacement of other proteins that are essential for telomere stability and replication (e.g., Pot1, TRF2, and helicases), and to follow the progression of replication forks at telomeres.

## 4. Materials and Methods

### 4.1. Cell Culture and Cell Growth Experiments

A549 cells were purchased from ATCC and cultured with Dulbecco’s modified Eagle’s medium (DMEM) with glutamax, supplemented with 10% fetal calf serum (FCS) and 1% penicillin/streptomycin. Cells were maintained in humidified incubators at 37 °C in an atmosphere containing 5% CO_2_ and ambient oxygen of 20%. Cells were grown in 25 cm^2^ flasks (150,000 cells/flask) and treated with 5 μM of 360A or (360A)_2A_ every 3 or 4 days (i.e., at each reseeding) until cultures were terminated. Control cells were either non-treated or treated with 0.1% DMSO. At each reseeding the remaining cells were pelleted and snap frozen for further DNA extraction.

### 4.2. DNA Extraction and STELA

DNA extraction was carried out with the ENZA tissue DNA kit for dry pellets of more than 100,000 cells and with the Qiagen QIAamp DNA mini kit (Les Ulis, France) for dry pellets of less than 100,000 cells. For 17p telomere length analysis we used the modified STELA protocol previously described [5,8]. The genomic DNA was diluted at 30 ng/μL in 10 mM Tris-HCl, pH 8. Then 120 ng of DNA was further diluted in 40 μL of 10 mM Tris-HCl, pH 8, containing 0.25 μM Telorette 2. Multiple PCRs were carried out for each diluted DNA sample in a 10 μL reaction mixture containing 3 ng of diluted DNA, 0.5 μM telomere adjacent (17p) and Teltail primers, 1X Taq Buffer and 0.5 U of a 10:1 mixture of Taq (Thermo Fisher, Courtaboeuf, France), and Pwo polymerase (Roche, Meylan, France). The reactions were cycled with a BioRad C 1000 touch thermocycler (Marnes la Coquette, France) under the following conditions: 22 cycles of 94 °C for 20 s, 59 °C for 30 s and 68 °C for 8 min. The DNA fragments were resolved by 0.5 % Tris-acetate-EDTA (TAE) agarose gel electrophoresis, and were detected by southern hybridization with a random-primed α-^33^P-labeled (Perkin Elmer, Villebon, France) telomere probe and a probe to detect the 1 kb (Stratagene, Les Ulis, France) and 2.5 kb (BioRad) molecular weight marker. The hybridized fragments were detected by phosphorimaging with a Thyphoon FLA 9500 (GE healthcare, Velizy, France). The molecular weights of the DNA fragments were calculated using Total Lab Quant (Newcastle, UK).

### 4.3. Oligonucleotides

17pseq1rev: 5′-GAATCCACGGATTGCTTTGTGTAC-3′

Teltail: 5′-TGCTCCGTGCATCTGGCATC-3′

Telorette 2: 5′-TGCTCCGTGCATCTGGCATCTAACCCT-3’

### 4.4. Chemical Compounds

360A and (360A)_2A_ were synthesized as previously described [26]. Stock solutions were prepared in dimethyl sulfoxide (DMSO) at a concentration of 5 mM and stored at −20 °C.

### 4.5. Statistical Analysis

Statistical analysis was performed using GraphPad Prism 5. A Chi-square test was used to compare TDEs’ frequencies.
